# Angiogenic Factor Expression in Hepatic Cirrhosis

**DOI:** 10.1155/2007/67187

**Published:** 2007-02-28

**Authors:** Alexandra Giatromanolaki, Stamatia Kotsiou, Michael I. Koukourakis, Efthimios Sivridis

**Affiliations:** ^1^Department of Pathology, Democritus University of Thrace Medical School, P.O. Box 12, 68100 Alexandroupolis, Greece; ^2^First Department of Medicine, Democritus University of Thrace Medical School, P.O. Box 12, 68100 Alexandroupolis, Greece; ^3^Department of Radiotherapy/Oncology, Democritus University of Thrace Medical School, P.O. Box 12, 68100 Alexandroupolis, Greece

## Abstract

The pathogenesis of fibrosis in hepatic cirrhosis remains obscure. This study examines the eventual role of angiogenic factors in the fibrotic process. A series of 55 cirrhotic livers was studied for the proliferation state of fibroblasts, and the expression of vascular endothelial growth factor (VEGF), thymidine phosphorylase (TP) and the basic and acidic fibroblast growth factor (bFGF, aFGF) in both fibroblasts and hepatic cells. The angiogenic and/or fibrogenic factors VEGF, TP, bFGF, and aFGF were clearly expressed in regenerative hepatocytes, but not in fibroblasts of diffuse hepatic fibrosis. The immunohistochemical findings suggest that angiogenic factors and factors promoting oxidative stress (i.e., TP) produced by hepatocytes may contribute to the development of fibrous bands in hepatic cirrhosis.

## 1. INTRODUCTION

Cirrhosis is characterized by diffuse hepatic fibrosis, in the form of delicate bands or broad scars, replacing the normal lobular architecture and encompassing regenerative nodules of hepatocytes. Most cases of cirrhosis are attributable to alcoholic liver disease and chronic viral hepatitis, while less frequent causes include autoimmune hepatitis, biliary disease, drugs, hemochromatosis, Wilson's disease, *α*_1_-antitrypsin deficiency, and galactosemia and tyrosinemia in infants and children.

The molecular events leading to fibrotic process remain, by and large, obscure in hepatic cirrhosis. The highly vascularized fibrous tissue, surrounding the regenerative hepatic nodules, suggests that angiogenic factors may be involved in the pathogenesis of the disease. Experimental data support such a hypothesis and angiogenic factors expressed by hepatocytes have been implicated as a key event in the development of hepatic fibrosis [1–3].

In the current study, we investigated the expression of angiogenic and fibrogenic growth factors in cirrhotic livers, providing evidence that the overproduction of these factors by hepatocytes, but not stromal cells, may play an important role in the pathogenesis of the disease.

## 2. MATERIALS AND METHODS

Formalin-fixed paraffin-embedded tissues from biopsies of 55 patients with fully developed micronodular cirrhosis were retrieved from the archives of the Department of Pathology, Democritus University of Thrace Medical School, Alexandroupolis, Greece. All cases were of a posthepatitic etiology for the patients having a long-standing history of chronic viral hepatitis B with a histological activity index ranging from 11 to 18. Furthermore, the cirrhotic livers were characterized by the presence of an ongoing necroinflammation, mainly in the form of piecemeal necrosis. There was, however, no evidence of large-cell or small-cell liver cell dysplasia and no indication of hepatocellular carcinoma.

A standard immunohistochemical technique, with the appropriate antibodies and controls, was applied (a) to assess the proliferation state of fibroblasts and (b) to detect the expression of various angiogenic and fibrogenic factors—the vascular endothelial growth factor (VEGF), the thymidine phosphorylase (TP), and the basic and acidic fibroblast growth factors (bFGF, aFGF). Details of the immunohistochemical techniques [4, 5] and the primary antibodies used are shown in Table 1.

Immunohistochemical evaluation was performed by two observers (GA, SE) over the conference microscope. The extent (diffuse versus focal) and the intensity (strong versus weak versus absent) of the cytoplasmic and/or nuclear expression of the proteins analyzed were recorded at ×200 magnification.

## 3. RESULTS

Basic fibroblast growth factor (bFGF), aFGF, and VEGF were expressed diffusely and uniformly in the cytoplasm of hepatocytes throughout the entire cirrhotic liver. In all cases, the intensity of staining was weak, but definitely present (in 100% of cases examined). In contrast, the adjacent stromal fibroblasts and hepatocytes from normal liver samples were persistently negative. Occasionally, small blood vessels were positively stained.

Thymidine phosphorylase (TP), a marker of oxidative stress, was expressed strongly to a varying extent by hepatocytes. Nuclear and cytoplasmic expression was noted in 42 out of 55 cases, ranging between 5%–80% of cells examined. In 19 out of 55 (34.5%) cases, the TP expression in hepatocytes was prominent (more than 50% of hepatocytes were stained). Again, the stroma was negative. Hepatocytes from normal liver showed a weak staining for TP.

The MIB1 proliferation index was of a very low proliferation activity in fibroblasts. Staining was noted in 14 out of 55 cases, with a percentage of positivity not exceeding 2%. Similarly, proliferating activity of hepatocytes was low, ranging from 0% to 5%. MIB1 staining was noted in 22 out of 55 cases (40%), where 6 out of 55 (11%) exhibited a relatively high expression (5% of the total hepatocyte population examined).

There was no association of patterns of expression among the molecular features examined, nor with MIB1 proliferation index.

**Figure 1:**
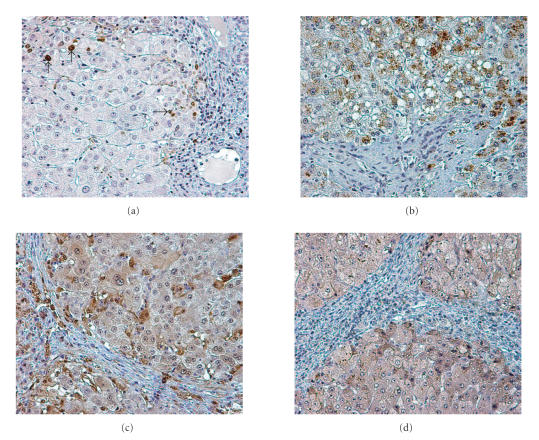
Immunohistchemical study of liver cirrhosis: (a) MIB1 nuclear staining in hepatocytes; (b) granular cytoplasmic expression of bFGF in hepatocytes; (c) thymidine phosphorylase expression in the cytoplasm and nuclei of hepatocytes; (d) cytoplasmic expression of VEGF in hepatocytes. The adjacent fibrous bands do not express any of these proteins.

Figure 1 shows characteristic immunostaining images indicating the expression patterns of the above proteins.

## 4. DISCUSSION

There is experimental evidence that VEGF plays an important role in the development of hepatic cirrhosis. Rosmorduc et al., using rats as experimental models, indicated that biliary cirrhosis is associated with hepatocellular hypoxia [2]. VEGF is known to be induced under hypoxic conditions, as a result of a transcriptional activation of the VEGF gene mediated by the hypoxia-driven increased HIF1*α* protein accumulation [6]. Rats treated with diethylnitrosamine, an agent inducing chemical cirrhosis, show progressive liver fibrosis accompanied by increased expression of VEGF and VEGF-receptor and active angiogenesis [1] Similarly, Yoshiji et al., using an experimental model of chemical induction of hepatic cirrhosis, associated the development of liver fibrosis with a significant increase of VEGF mRNA expression in the liver [7]. Administration of neutralizing monoclonal antibodies against VEGF receptors suppressed angiogenesis and significantly reduced the development of fibrosis.

The above experimental evidence is confirmed in our histopathological study, as VEGF was overexpressed in cirrhotic hepatocytes compared to normal liver cells. This finding is also in accordance with Shi's et al. recent report [8]. In another study by Li et al., VEGF levels, measured in the plasma of patients and healthy controls, showed a 1.5- fold increase in cirrhotic patients [9]. Spider angiomas, frequently noted in cirrhotic patients, were more frequent in patients with high VEGF plasma levels, consistent with the VEGF angiogenic activity.

The expression of both acidic and basic fibroblast growth factors was also found increased in regenerative cirrhotic hepatocytes. The importance of these factors in the development of the disease has been previously proposed in the study of Li et al., where high bFGF plasma levels paralleled high VEGF levels in cirrhotic patients [9]. bFGF has a broad range of activity on both hepatocytes and stromal cells via specific receptors, as shown by Huang et al. [10]. A potent synergy of VEGF and bFGF receptors activation in inducing angiogenesis was noted. Using aFGF and bFGF deficient mice, Yu et al. showed that liver fibrosis, resulting from chronic exposure to carbon tetrachloride, was dramatically decreased in these mice compared to controls [3].

In contrast to hepatocytes, fibroblasts were totally unreactive to the above angiogenic factors. Moreover, the proliferation index, as assessed with the MIB1 monoclonal antibody, was very low. These findings show that the fibrotic process, as it occurs in the context of cirrhosis, represents a slow fibroblastic response to external stimuli, that is, VEGF and FGF, produced by hepatocytes. Altered fibroblast biology, through activation of such genes, does not seem to contribute to the process. This suggestion is further supported by the absolute lack of expression of thymidine phosphorylase in fibroblasts, a marker of DNA synthesis and of oxidative stress [11]. TP is frequently upregulated in actively proliferating fibroblasts, that is, in the context of neoplasia [12, 13].

It is concluded that growth factors such as VEGF, aFGF, and bFGF produced by hepatocytes in patients with liver cirrhosis may have an important role in the development of hepatic fibrosis through progressive stimulation of fibroblasts.

## Figures and Tables

**Table 1:** Details of the antibodies, dilutions, and antigen retrieval methods used in this study, MW = microwave heating.

Primary antibody	Dilution/incubation time	Antigen retrieval	Specificity	Source

VG1	1 : 4 (75 min^a^)	MW	VEGF	Oxford University
P-GF.44C	1 : 4 (75 min^a^)	MW	TP	Oxford University
FGF-2 (147): *sc-79*	1 : 100 (75 min^a^)	MW	bFGF	Santa Cruz Biotechnology, Inc.
FGF-1 (C-19): *sc-1884*	1 : 100 (75 min^a^)	MW	aFGF	Santa Cruz Biotechnology, Inc.
MIB1	1 : 75 (75 min^a^)	MW	Ki-67 antigen	DAKO, Glostrup, Denmark

^a^At room temperature.
